# Re-engineering cellular physiology by rewiring high-level global regulatory genes

**DOI:** 10.1038/srep17653

**Published:** 2015-12-03

**Authors:** Stephen Fitzgerald, Shane C. Dillon, Tzu-Chiao Chao, Heather L. Wiencko, Karsten Hokamp, Andrew D. S. Cameron, Charles J. Dorman

**Affiliations:** 1 Department of Microbiology, Moyne Institute of Preventive Medicine, Trinity College Dublin, Dublin 2, Ireland; 2 Department of Biology, University of Regina, Saskatchewan, Canada; 3 Department of Genetics, Smurfit Institute, Trinity College Dublin, Dublin 2, Ireland

## Abstract

Knowledge of global regulatory networks has been exploited to rewire the gene control programmes of the model bacterium *Salmonella enterica* serovar Typhimurium. The product is an organism with competitive fitness that is superior to that of the wild type but tuneable under specific growth conditions. The paralogous *hns* and *stpA* global regulatory genes are located in distinct regions of the chromosome and control hundreds of target genes, many of which contribute to stress resistance. The locations of the *hns* and *stpA* open reading frames were exchanged reciprocally, each acquiring the transcription control signals of the other. The new strain had none of the compensatory mutations normally associated with alterations to *hns* expression in *Salmonella*; instead it displayed rescheduled expression of the stress and stationary phase sigma factor RpoS and its regulon. Thus the expression patterns of global regulators can be adjusted artificially to manipulate microbial physiology, creating a new and resilient organism.

Gene expression in bacteria is controlled by regulatory factors that include *trans*-acting DNA binding proteins that operate within hierarchies: the more global the influence of the regulatory protein, the higher its place in the hierarchy^1^,^2^,^3^,^4^. Any reorganization of the expression pattern of global regulators is likely to alter regulatory network topology and hence cellular physiology^3^,^4^,^5^,^6^,^7^. Nucleoid-associated proteins (NAPs) represent a class of global regulatory factor with a very wide range of influences in the genome. NAPs act at the nexus of gene regulation and nucleoid architecture and they typically govern the expression of hundreds of genes, usually in collaboration with other regulatory proteins^8^. The model bacteria *Escherichia coli* and its close relative *Salmonella enterica* serovar Typhimurium have at least 14 NAPs, several of which form paralogous pairs^8^. Among these are the paralogues H-NS and StpA, proteins that directly or indirectly regulate 13% and 5%, respectively, of the genes in *S*. Typhimurium in two overlapping regulons^9^,^10^.

In *S.* Typhimurium, the amino acid sequences of H-NS and StpA are 58% identical and the proteins have many functional characteristics in common^9^,^10^,^11^. They also share numerous binding loci in the genome and are involved predominantly in silencing transcription, especially that of genes with a high A + T base composition^10^,^12^. Despite these structural and functional similarities, H-NS and StpA are distinguishable. The two proteins have different expression patterns: H-NS is expressed to a high level throughout the growth cycle while expression of StpA is lower and associated principally with the exponential phase of growth^13^,^14^. The *hns* and *stpA* genes also differ in their transcription regulation: the *hns* promoter is stimulated by the nucleoid-associated protein Fis^15^ and the cold-shock protein CspA^16^, is inhibited by the iron regulatory protein Fur^17^ and is subject to auto-repression by H-NS^18^. The *stpA* promoter is stimulated by osmotic shock and by increases in growth temperature; it shows a sustained expression in minimal medium that depends on the leucine-responsive regulatory protein, Lrp, which is lost upon carbon starvation; transcription of *stpA* is also silenced by H-NS^14^,^19^.

The *hns* and *stpA* genes also differ in their genomic locations: *hns* is in the Ter macrodomain of the chromosome close to the point where chromosome replication ends, making *hns* among the last genes on the chromosome to be duplicated at completion of the cell cycle^2^,^20^. In contrast, the *stpA* gene is in the non-structured region of the left replichore of the chromosome, mid-way between the origin and terminus of chromosome replication^20^. Gene location is known to be an important influence on expression in eukaryotes^21^,^22^,^23^ and this has recently been established in bacteria too^24^. To date, experiments that have investigated the effects of gene location on expression typically have involved moving the promoter of a gene of interest to a series of different locations and monitoring its expression pattern^25^,^26^,^27^,^28^,^29^,^30^,^31^,^32^,^33^. Interestingly, moving the entire *hns* gene together with its negatively-auto-regulated promoter and upstream regulatory region to different locations on the chromosome did not alter cell physiology in *E. coli*^26^. We decided to probe the matter of upstream sequence influences on paralogous gene regulation by taking a much more subtle approach in which just the open reading frames (ORFs) of the two highly-related *hns* and *stpA* genes were exchanged reciprocally on the *Salmonella* chromosome. Since the exchanges began at the translation initiation codons and ended at the translation stop codons, all other components of the participating genes – the transcription regulation, initiation and termination signals – remained in place and unchanged. This allowed us to investigate specifically the effects of ORF exchange while minimizing the number of other variables.

## Results

### Regulatory re-wiring of the *hns* and *stpA* open-reading frames

**Figure 1:**
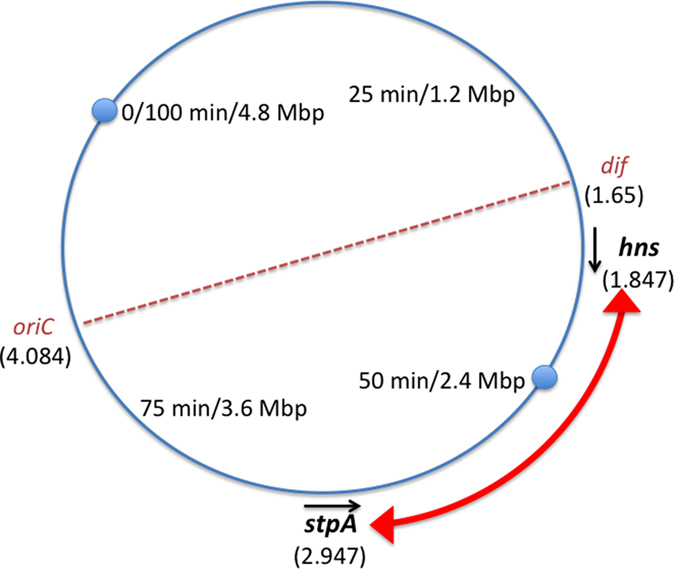
The chromosome of *Salmonella* divided into quarter segments calibrated in minutes and megabasepairs (Mbp). The locations of the origin of chromosome replication (*oriC*) and the site at the mid-point of the chromosome terminus at which chromosome dimers are resolved by site-specific recombination (*dif*) are indicated. The locations and directions of transcription of the *stpA* and *hns* genes are shown. The numbers in parentheses are the coordinates (in Mbp) of the first nucleotides of their open reading frames; the zero reference point is at 100 minutes (4.8 Mbp). The chromosome is bi-directionally replicated from the origin to the terminus and in *Salmonella* the *stpA* and *hns* genes are both located in the left replichore.

To investigate the physiological significance of the distinct expression patterns of the *hns* and *stpA* genes, their ORFs were exchanged reciprocally on the chromosome of *S*. Typhimurium strain SL1344 (Methods). In the resulting strain, SL1344RX (RX: reciprocal exchange) the *hns* ORF was connected to the transcription regulatory inputs of *stpA* and *vice versa* (Fig. 1) (Fig S1 and S2). Whole genome sequencing revealed that SL1344RX had not acquired any of the compensatory mutations previously associated with loss of *hns* expression in SL1344^9^,^10^ (Table S1, Genome Accession Number CP011233).

### SL1344RX has fitness that is tuneable and can out-compete the wild type

**Figure 2:**
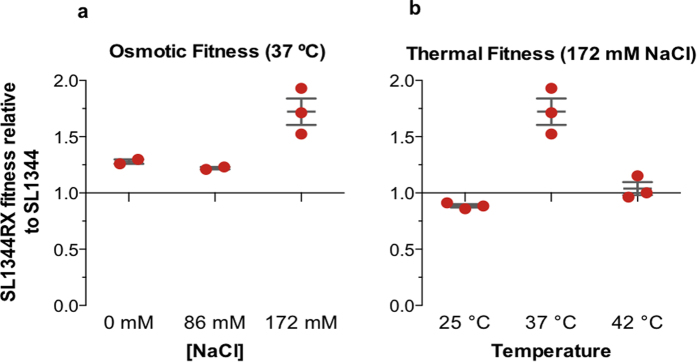
Competitive fitness of SL1344RX relative to SL1344 under different growth conditions. (**a**) Fitness of SL1344RX relative to SL1344 grown at 37˚C in a growth medium supplemented with 0 (N = 2), 86 (N = 2) or 172 (N = 3) mM NaCl. Relative fitness values above 1.0 for SL1344RX indicate greater fitness than SL1344; values below 1.0 indicate that SL1344RX is less fit than SL1344. (**b**) Fitness of SL1344RX relative to SL1344 at three growth temperatures: 25˚C, 37˚C and 42˚C (N = 3). Data points, their mean values and standard deviations are plotted for the biological replicates.

H-NS and StpA regulate genes required for adaptation to environmental stresses, most notably temperature and osmotic shifts^10^,^34^,^35^,^36^,^37^. Therefore we assessed the impact of re-wiring the *hns* and *stpA* loci on the competitive fitness of *S*. Typhimurium growing at different temperatures and osmotic pressures. This was done by competing co-cultured SL1344 and SL1344RX at 25 °C, 37 °C or 42 °C (Fig. 2a) and at 37 °C in L broth supplemented with 0 mM, 86 mM or 172 mM NaCl (Fig. 2b).

Re-wiring of the *hns* and *stpA* genes in SL1344RX altered competitive fitness in a temperature-specific manner. At 25 °C SL1344RX was less fit than the wild type (|s| = 0.88). In strong contrast, at 37 °C SL1344RX significantly out-competed the wild type (|s| = 1.72); no discernable difference in fitness relative to SL1344 was observed at 42 °C (Fig. 2). The fitness enhancement seen in SL1344RX at 37 °C could be tuned by varying the salt concentration of the growth medium: in NaCl-free broth a relative fitness index of 1.27 was recorded for SL1344RX and a marginal decrease in this value was recorded when the strains were competed in 86 mM NaCl (|s| = 1.22); the greatest fitness enhancement was seen at 172  mM NaCl at 37 °C (Fig. 2).

### *hns* and *stpA* exhibit new expression patterns in SL1344RX

**Figure 3:**
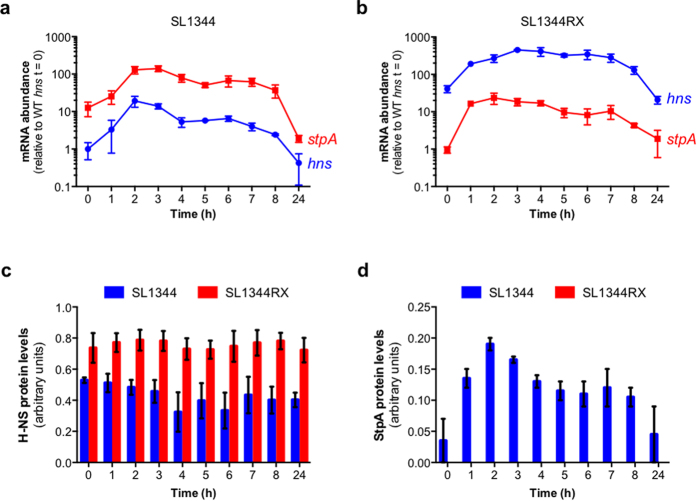
Expression of hns and stpA mRNA and protein in SL1344 and SL1344RX. Transcripts encoded by the *hns* (blue) and *stpA* (red) open reading frames in SL1344 (wild type) (**a**) and SL1344RX (**b**) as a function of time were monitored by RT-qPCR. Transcript levels are expressed relative to the level of the *hns* mRNA transcript in wild type SL1344 at t = 0. H-NS (**c**) and StpA (**d**) protein levels in SL1344 (blue) and SL1344RX (red) were determined by Western blotting. Protein levels were measured by densitometry and expressed relative to an internal control in arbitrary units. The mean and standard deviation for mRNA and protein levels are plotted for three biological replicates (N = 3).

The relocated *hns* and *stpA* ORFs did not retain their normal expression patterns: changes in the abundance of *hns* mRNA and *stpA* mRNA were observed in SL1344RX when compared to wild-type SL1344 (Fig. 3). When *hns* mRNA levels were monitored in wild-type SL1344 (Fig. 3a), a characteristic peak was observed during exponential growth after which mRNA levels gradually declined. In SL1344RX, where the *stpA* promoter drives transcription of the *hns* ORF, an *stpA*-like pattern of expression was not conferred upon *hns*. Instead, a much higher level of *hns* expression was detected in SL1344RX compared to the wild type (Fig. 3b).

The repositioned *stpA* ORF had also acquired a new pattern of expression in SL1344RX, peaking earlier in the growth cycle than in SL1344 (Fig. 3b). Maximum expression of *stpA* was sustained for longer in SL1344RX compared to the wild type (Fig. 3a). Despite being transcribed from the *hns* promoter, *stpA* mRNA was on average 6-fold less abundant in SL1344RX than in SL1344 throughout the growth cycle.

As the ORF exchanges in SL1344RX altered both the timing and abundance of *hns* and *stpA* mRNA expression, it was anticipated that these exchanges could also change the relative sizes of the H-NS and StpA protein populations. Therefore, H-NS and StpA protein expression was monitored as a function of the growth cycle in SL1344 and SL1344RX by western blotting.

In agreement with previously published data^18^, the abundance of H-NS protein did not change over the growth cycle in wild-type SL1344 (Fig. 3c). H-NS was similarly expressed at a constant level throughout the growth cycle in SL1344RX, despite the new pattern of mRNA expression from the *stpA* promoter. However, when the levels of H-NS protein in each strain were quantified, it was discovered that H-NS was up to 2-fold more abundant in SL1344RX than in SL1344 (Fig. 3c).

The abundance of StpA protein in wild-type SL1344 agreed with published data^13^ (Fig. 3d). Contrary to the comparable levels of H-NS in the wild type and SL1344RX, no StpA protein could be detected at any stage of growth in SL1344RX (Fig. 3d). The observation that *stpA* mRNA levels were lower in SL1344RX than in the wild type raised the possibility that StpA concentrations may have been too low to detect by Western blotting. When total protein from SL1344 and from SL1344RX was analysed by mass spectrometry, StpA-specific peptides were detected in SL1344 but not in SL1344RX (Supplementary Information), consistent with an absence of StpA in SL1344RX.

As mRNA from the *stpA* ORF was readily detectable in SL1344RX (Fig. 3), transcription silencing by H-NS seemed an unlikely explanation for the lack of StpA protein. Repositioning the *hns* and *stpA* open reading frames had the inevitable consequence of creating novel transcripts consisting of the untranslated region (UTR) of one gene fused to the other gene’s ORF. We tested whether these hybrid molecules, 5′-*hns[UTR]-stpA[ORF]*-3′ mRNA and 5′-*stpA[UTR]-hns[ORF]*-3′ had different stabilities compared to wild-type transcripts.

**Figure 4:**
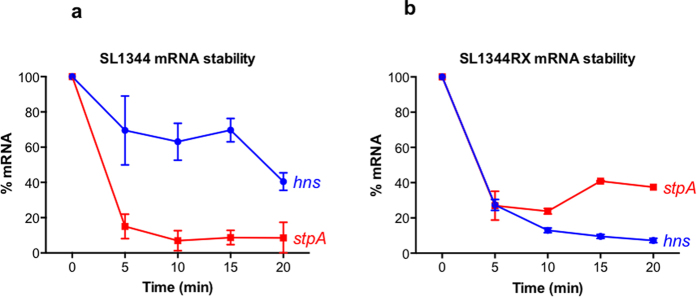
Relative stability of mRNA in SL1344 (wild type) and SL1344RX. The levels of *hns* mRNA and *stpA* mRNA were monitored at regular intervals (0, 5, 10, 15 and 20 min) post-rifampicin treatment in SL1344 (**a**) and SL1344RX (**b**).

The stabilities of the 5′-*stpA[UTR]-hns[ORF]*-3′ and 5′-*hns[UTR]-stpA[ORF]*-3′ hybrid mRNAs in SL1344RX were compared to native transcripts in SL1344 over a 20-min time course by RT-qPCR following transcription inhibition by rifampicin treatment (Methods). The results showed that 5′-*stpA[UTR]-hns[ORF]*-3′ was less stable than native *hns* mRNA (Fig. 4a,b) despite generating elevated levels of H-NS in SL1344RX (Fig. 3). In contrast, the hybrid 5′-*hns[UTR]-stpA[ORF]*-3′ mRNA in SL1344RX was *more* stable than native *stpA* mRNA in SL1344 (Fig. 4a,b). Therefore reduced message stability does not account for the absence of StpA protein in SL1344RX. The translatability of the hybrid 5′-*hns[UTR]-stpA[ORF]*-3′ message and its susceptibility to modulation by RNA secondary structure formation were not measured directly and may underlie the strong reduction in StpA protein in SL1344RX.

### RpoS expression is rescheduled in SL1344RX

**Figure 5:**
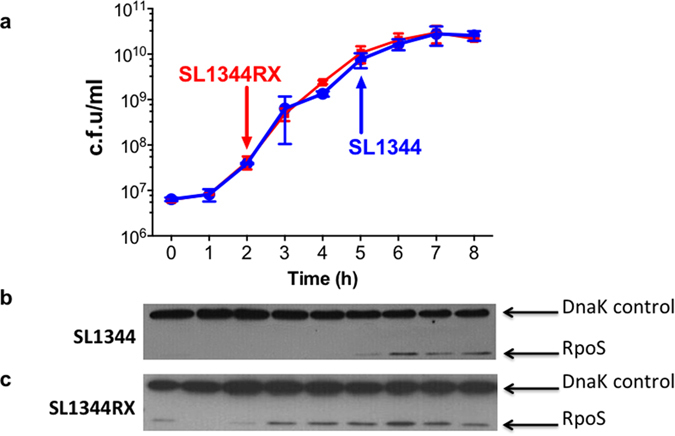
Expression of the RpoS sigma factor in SL1344 (wild type) and in SL1344RX as a function of growth. The growth cycles of SL1344 and SL1344RX were determined by colony counts (**a**). RpoS expression was monitored by Western blotting and was first detected after 2 h and 5 h of growth in SL1344 (**b**) and SL1344RX (**c**), respectively.

The transcriptomes of SL1344RX and SL1344 were compared during mid-exponential growth (OD_600_ = 0.3) by gene expression microarray analysis. Despite the involvement of H-NS and StpA in regulating so many genes, we identified only 26 differentially expressed genes in SL1344RX relative to SL1344 during exponential growth (Table S2). Twenty-three of these genes were up-regulated and 3 down-regulated in SL1344RX, and 13 were predicted bioinformatically to be RpoS-dependent (Table S3). Further analysis of H-NS, StpA and RpoS regulated genes revealed subtle but significant (P < 0.01) changes in expression in comparison with control gene sets across both the RpoS and StpA regulons but not the H-NS regulon (Fig. S3). Loss of H-NS was shown previously to result in compensatory mutations in the *rpoS* gene and inactivation of *stpA* expression to alter the timing of RpoS expression^9^,^10^. We detected no compensatory mutations but did find that RpoS was expressed in exponential phase in SL1344RX (Fig. 5) and at a much earlier time than in the *stpA* knockout derivative of SL1344^10^.

Many genes within the RpoS regulon are required for adaptation and resistance to environmental stresses such as oxidative, osmotic and thermal stress^38^,^39^,^40^. Up-regulation of RpoS regulon members could explain the enhanced fitness of SL1344RX at 37 °C under different degrees of osmotic pressure (Fig. 2b). Consistent with this model, many of the up-regulated RpoS-dependent genes in SL1344RX were involved in osmotic stress resistance (e.g. *osmE*, *osmY*, *otsB, wrbA, ybaY*, *yehY*, and *ygaU*).

Expression of RpoS-regulated genes (and RpoS itself) is normally confined to periods of stress and to stationary phase^40^,^41^. Inappropriate expression of the RpoS regulon in *E. coli* has been described as detrimental to competitive fitness because RpoS competes with other sigma factors for core RNA polymerase resulting in decreased expression of RpoS-independent genes involved in nutrient uptake and metabolism^42^,^43^,^44^,^45^. The cellular concentration of RpoS is therefore tightly regulated at the level of *rpoS* transcription, translation and proteolytically at the post-translational level^39^.

In *S*. Typhimurium and *E. coli*, both H-NS and StpA modulate the proteolysis of RpoS by repressing expression of the IraM, IraD and RssC anti-adaptor molecules that prevent degradation of RpoS by the ClpXP protease^10^,^38^,^42^. Since the change in expression of RpoS regulon members in SL1344RX could have been due to a novel pattern of RpoS expression, we monitored RpoS abundance in SL1344 and SL1344RX as a function of growth stage. In the wild-type strain SL1344, RpoS was detectable, as expected, at the onset of stationary phase, 5 h post-inoculation (Fig. 5a,b); however RpoS appeared in SL1344RX in the early stages of exponential growth, 2 h post-inoculation, i.e. 3 h before detection in the wild type (Fig. 5a,c).

SL1344RX did not express measurable levels of StpA protein, although the 5′-*hns-stpA*-3′ mRNA was detectable. A knockout mutation in the *stpA* gene in SL1344 causes RpoS to appear in exponentially growing cells^10^ although not as early as in SL1344RX (Fig. 5c). Further evidence that the observed rescheduling of RpoS expression in SL1344RX was not simply correlated with the absence of StpA came from monitoring RpoS expression in a derivative of SL1344 with two *stpA* ORFs, one in its native location and one in place of *hns*; here, RpoS became detectable at 3 h post-inoculation (rather than at 2 h, as in SL1344RX). In yet another derivative of SL1344 that had two *hns* ORFs, one at its native location and one in place of *stpA,* RpoS appeared at 4 h post-inoculation (Fig. S4). These findings indicated that the timing of RpoS expression in growing SL1344 was modulated by the overall composition of the StpA and H-NS protein population in the different *hns/stpA* genotypes and not simply the presence/absence of StpA.

## Discussion

Synthetic biology and the creation of artificial gene regulatory circuits are contributing to a deeper understanding of the modular and hierarchical structures of complex regulatory networks^5^,^46^. This understanding is being exploited for the rational design and engineering of microbes for use in a wide range of industrial, biotechnological and biomedical processes^46^,^47^. Examples include the recent application of re-engineered strains of *E. coli* as biosensors for the detection of cancer and diabetes in mice^48^,^49^. The engineering of stress-tolerant microbes for industrial use has also been a major focus of synthetic biology^47^.

Our study shows that it is possible to generate a novel derivative of a well-characterized model bacterium that has improved competitive fitness by a simple reciprocal exchange of two paralogous genes that encode global regulators of gene expression. This approach permits retuning of existing gene regulatory networks and is complementary to bottom-up approaches to strain construction and synthetic biology based on the creation of artificial chromosomes. It also informs these approaches by adding to knowledge of the significance of gene position and expression for the preservation of genome integrity^50^,^51^. Because compensatory mutations were not detected in the SL1344RX genome sequence, the reciprocal genetic exchange that produced SL1344RX is well tolerated. The enhanced fitness of SL1344RX raises the question of why this genetic arrangement has not occurred in nature, perhaps as a result of horizontal gene transfer? First, it cannot be ruled out conclusively that strains similar to SL1344RX do not exist naturally; they just have not been reported before. Second, our study identified growth conditions (growth at 25 °C) where SL1344RX is outcompeted by the wild type. Thus, the rewired strain may not be capable of thriving in the external environment where it will quickly be eliminated by natural selection.

The RpoS stress and stationary phase sigma factor is expressed prematurely in the growth cycle of SL1344RX. This component of RNA polymerase is central to adaptation to osmotic and thermal stress and is a determining factor in bacterial fitness^40^,^41^,^42^,^44^,^45^. Both H-NS and StpA are intimately involved in the regulatory pathways that control RpoS expression and degradation^10^,^38^,^52^, so the early appearance of RpoS in SL1344RX is perhaps not altogether surprising. However, as we have not identified the molecular connection between RpoS and the *hns* and *stpA* genes that is responsible for altered RpoS expression in SL1344RX, it is possible that this involves an indirect effect. It is also important to note that since knockout mutations in *stpA* have obvious phenotypes in *Salmonella*^10^ but cause more subtle effects in *E. coli*^19^, it is probably unwise to extrapolate our findings even to closely related bacterial species.

The regulatory changes made to *hns* and *stpA* in SL1344RX altered the fitness of *S*. Typhimurium in an environment-specific manner. The variability of fitness outcomes between environmental conditions suggested that altering the relative expression of *hns* and *stpA* is one mechanism by which *S*. Typhimurium manipulates expression of genes within the combined H-NS/StpA regulatory network to adapt to environmental changes. Perhaps unsurprisingly, *hns* and *stpA* expression is naturally sensitive to temperature shifts: in *E. coli*, *hns* is up-regulated in response to cold-shock while up-regulation of *stpA* occurs upon an upshift in temperature from 30 °C to 37 °C^14^,^16^,^39^. Expression of *stpA* in *E. coli* is also strongly up-regulated in response to osmotic shock^16^. The ‘unnatural’ changes to the expression of *hns* and *stpA* brought about by re-engineering in SL1344RX conferred significant fitness advantages in certain environments, highlighting the potential of *S*. Typhimurium to evolve to be more competitive through the accumulation of mutations or the acquisition or loss of regulatory inputs that effect the expression of *hns* and *stpA*. It is also important to consider the impact of additional *hns-*like genes that are acquired horizontally; genes of this type are found on many mobile genetic elements that are capable of either self-transmission or of being mobilized^53^,^54^.

Conventionally a bacterial species has been viewed as a manifestation of defining phenotypes, of the presence of signature genes, and of the outcome of DNA sequence comparisons with reference strains^55^. Genomics is enriching this view by revealing that non-coding regions of DNA have a profound influence on species character. For example, orthologous genes with identical open reading frames can differ markedly in their regulatory regions so that the same genes can be used by different species to access different niches^56^,^57^. Thus, variation in non-coding regions offers a fruitful route to the evolution of new cellular properties by altering gene expression patterns^56^. The present study provides a dramatic example of the application of this approach to the construction in the laboratory of a bacterial strain with strikingly novel properties by a simple rewiring of two paralogous global regulators.

## Methods

### Bacterial strains and growth conditions

Bacterial strains used in this study, their genotypes and source, where applicable, are listed in Table S4; all are derivatives of *S*. Typhimurium strain SL1344. Unless otherwise stated bacterial strains were cultured with aeration (200 rpm) in Luria broth (LB) at 37 °C. Kanamycin, tetracycline, carbenicillin and chloramphenicol were used at concentrations of 50 μg ml^−1^, 15 μg ml^−1^, 50 μg ml^−1^ and 20 μg ml^−1^ respectively.

### Strain construction and DNA manipulation

All strains generated during this study were constructed using bacteriophage-lambda-mediated recombination^58^. To construct SL1344RX the *neoR* and *tetRA* resistance genes were PCR amplified from plasmids pSUB11^59^ and pACYC184. These amplicons were integrated onto the SL1344 chromosome 40 bp downstream of the *hns* and *stpA* stop codons, respectively. The *hns* ORF and downstream *neoR* gene were then PCR amplified and the resulting amplicon was integrated onto the SL1344 chromosome replacing the *stpA* ORF. This created a strain with two *hns* ORFs (SL1344^2X*hns*^). Similarly the *stpA* ORF and *tetRA* cassette were PCR amplified and used to replace the *hns* ORF, creating a strain with two *stpA* ORFs (SL1344^2X*stpA*^). The P_*hns*_-*stpA* promoter-ORF fusion from SL1344^2X*stpA*^ was then introduced into SL1344^2X*hns*^ by P22 phage-mediated generalised transduction^60^ to produce SL1344RX. Strain variants of SL1344 and SL1344RX expressing 3X FLAG-epitope-tagged H-NS or StpA were constructed using a modified version of bacteriophage-lambda-mediated recombination^60^. The DNA oligonucleotides used in this study and their uses are listed in Table S5.

### Real-time PCR

The *hns* and *stpA* mRNA levels in SL1344 and SL1344RX were determined by RT-qPCR. 0.2 OD_600nm_ units of bacteria were harvested, held on ice and 2/5 volume of stop solution (95% ethanol/5% phenol) was added. Total RNA was extracted using a Promega SV Total RNA Isolation kit and RNA concentration was quantified using a Nanodrop ND-1000. Extracted RNA (200 ng) was reverse transcribed using GoScript™ reverse transcription system (Promega) to generate cDNA pools and the relative abundances of target mRNA molecules were determined by quantitative PCR (qPCR) using gene specific primer pairs (Table S5) and GoTaq qPCR master mix (Promega). Quantification of mRNA was achieved using an internal calibration curve generated from serially diluted genomic DNA of known quantity. For mRNA stability assays, cultures were grown to an OD_600_ = 0.2 before the addition of rifampicin (250 μg ml^−1^) to inhibit further transcription. Levels of mRNA were then quantified at 0, 5, 10, 15 and 20 min after rifampicin addition.

### Western immunoblotting

Bacteria (0.2 OD_600 nm_ units) were harvested by centrifugation (13,000 rpm, 5 min) and re-suspended in 1× Laemmli sample buffer^61^. 12% SDS-polyacrylamide gels were used for SDS-PAGE and separated proteins were electroblotted onto 0.22 Protran nitrocellulose membranes (Whatman) for immunodetection as previously described^61^. Antibodies were used at the following dilutions: anti-FLAG (1/10,000) (Sigma), anti-RpoS (1/5000) (Neoclone) and anti-DnaK (1/100,000) (Abcam), secondary HRP-conjugated goat anti-mouse antibody (1/10,000). The Pierce West Pico Super Signal kit was used for chemiluminescent detection of bound peroxidase conjugate. Blots were probed for the presence of DnaK to ensure equal loading in each lane. Protein levels were quantified relative to a known concentration of Carboxy-Terminal FLAG-BAP fusion protein (Sigma) loaded in each gel.

### Competitive fitness assays

The fitness of SL1344RX relative to wild-type SL1344 was calculated for several environmental conditions as described previously^62^,^63^. Colony-forming units of each competing strain were enumerated at time zero (t = 0) and after 24 h of co-culturing by selective plating. The fitness/selection coefficient, |s|, of SL1344RX was calculated using the formula:


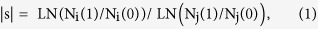


where N_i_ (0) and N_i_ (1) = initial and final colony counts of SL1344RX, respectively and N_j_ (0) and N_j_ (1) = initial and final colony counts of wild type SL1344, respectively^63^.

### RNA extraction, DNA labelling and hybridisation for microarray

OD_600 nm_ units of bacterial cultures were harvested during mid-exponential growth (OD_600 nm_ = 0.3). Total RNA was extracted using a Promega SV Total RNA Isolation kit, quantified using a Nanodrop ND-1000 and checked for purity and degradation by gel electrophoresis. Total RNA samples were converted to double-stranded cDNA pools using a Superscript™ Double-Stranded cDNA synthesis kit (Invitrogen). Three independent RNA samples were extracted for microarray analysis of transcript abundance. Double stranded cDNA (200 ng) was fluorescently labelled with Cy3™ dCTP using the BioPrime® Array CGH Labelling system (Invitrogen). 200 ng of isolated *S*. Typhimurium SL1344 genomic DNA was labelled with Cy5™ dCTP for use as a co-hybridization control and reference on all arrays. Labelled DNA and cDNA was purified using Micro-spin G50 columns (GE Healthcare) to remove unlabelled nucleotides. Cy3™ and Cy5™ labelled cDNA and reference genomic DNA were mixed in a 1:1 ratio (180 μl of each) and the combined DNA was ethanol precipitated. Precipitated pellets were re-suspended in 100 μl of hybridisation buffer (1 M NaCl, 100 mM MES pH 6.5, 20 mM EDTA, 20% formamide, 20% Triton X-100) at 70 °C for 15 min. Samples were then denatured (100 °C, 10  min), applied to a 44k-probe *Salmonella* Typhimurium SL1344 microarray slide (Oxford Gene Technologies) and allowed to hybridise for 48 h at 55 °C.

### Microarray data acquisition and analysis

After hybridisation, slides were washed and scanned with a GenePix 4000B scanner (Axon Instruments). Quantification of fluorescent spot intensities and local background data was performed using the GenePix 3.0 software supplied. Microarray data files were loaded into ArrayPipe^64^ for quality assessment and for normalisation with the loess function applied to each sub-grid. Multiple probes per gene were merged into one average value using the median. This was followed by between-array normalisation using the Rquantile method and differential expression analysis through the Bioconductor package Limma^65^. P-values were corrected for multiple testing through the Benjamini-Hochberg method. To detect subtle regulon-wide changes in gene expression, the expression intensities and Log_2_ ratios of known H-NS, StpA or RpoS regulated genes were used to generate MA-plots (Fig. S3). Gene expression across each regulon was compared with an equivalent number of control genes. Ten independent comparisons were made using randomly selected control gene sets and analysed by t-test to detect significant changes in expression (P < 0.01). The complete datasets are available at GEO (Accession number GSE52235).

### Genome sequencing and analysis

Whole genome sequencing was performed on an Illumina MiSeq platform. Genomic DNA was extracted using a Gentra® Puregene® Yeast/Bact. Kit (Qiagen) and quantified by Qubit dsDNA BR Assay (LifeTechnologies). A Bioruptor Standard (Diagenode, NJ, USA) was used to shear DNA (1 μg in 100 μl Tris-HCl, pH 7) by sonication (low power, 30 s on/90 s off, 15  min) to an optimal fragment size of 600–800 bp. NEBNext Ultra DNA library prep and NEBNext Multiplex Oligo kits for Illumina (New England Biolabs) were used to generate paired–end libraries. Libraries were quantified with the Qubit dsDNA HS assay (LifeTechnologies) and the quality and size distribution was assessed on an Agilent high sensitivity DNA chip with a Bioanalyzer (Agilent Technologies, CA, USA). A final combined 4 nM library was prepared, denatured, diluted to 14 pM and sequenced using a MiSeq Reagent v3 Kit (600 cycle) according to manufacturer guidelines (Illumina Inc., CA, USA).

Paired-end reads were first quality checked with FastQC and then assembled using the velvet *de novo* assembler, version 1.2.07^66^. Under default settings we varied k-mer and found k = 227 to produce the highest N50 of 377, 806. This generated 27 contigs of length greater than 1000  bp with the longest at 635, 541 bp. The contigs were ordered and concatenated using ABACAS version 1.3.1.2^67^ providing a full genome of length 4.96 Mbp. This assembly was then aligned to wild type *Salmonella* Typhimurium SL1344. The Geneious R8.1 package (Biomatters, Auckland, New Zealand)^68^ was used to align paired-end reads to the SL1344 reference genome (NC_016810, NC_017718, NC_017719, NC_017720). Aligned reads were then searched for variants with a minimum 95% frequency in reads and again at a relaxed stringency of 45% variant frequency. The quality of read alignment at variants was confirmed visually. Only two SNPs were detected and these were in genes (*menC* and *manX*) that have no known connections to H-NS, StpA or RpoS.

## Additional Information

**How to cite this article**: Fitzgerald, S. *et al.* Re-engineering cellular physiology by rewiring high-level global regulatory genes. *Sci. Rep.* **5**, 17653; doi: 10.1038/srep17653 (2015).

## Supplementary Material

Supplementary Information
