# Supplementary material for: Re-engineering cellular physiology by rewiring high-level global regulatory genes
Source: Sci Rep. 2015 Dec 3;5:17653. doi: 10.1038/srep17653 (PMC4668568; doi:10.1038/srep17653)
Supplement: Supplementary Information [file srep17653-s1.pdf]

**Re-engineering cellular physiology by rewiring high-level global regulatory genes**

Stephen Fitzgerald[1,2,], Shane C Dillon[1], Tzu-Chiao Chao[2], Heather L Wiencko[3], Karsten Hokamp[3], Andrew DS Cameron[2], Charles J Dorman[1]*

[1]Department of Microbiology, Moyne Institute of Preventive Medicine, Trinity College Dublin, Dublin 2, Ireland
[2]Department of Biology, University of Regina, Saskatchewan, Canada
[3]Department of Genetics, Smurfit Institute, Trinity College Dublin, Dublin 2, Ireland

Supplementary information

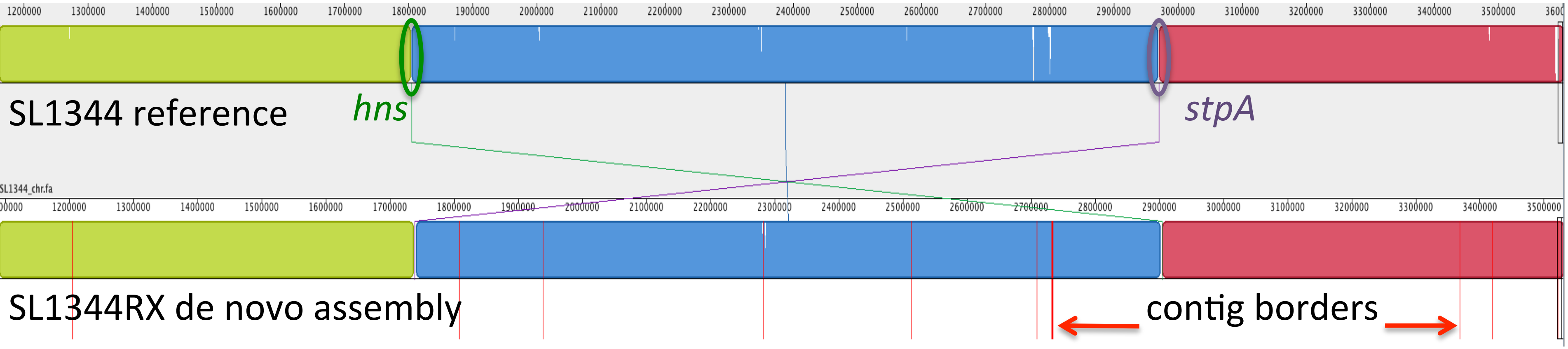


Figure S1| Visual alignment with Mauve, showing the wild type SL1344 genome (top) and SL1344RX (bottom). Aligned coloured blocks represent regions of homologous sequence that are free from genomic rearrangement. Strain SL1344RX aligns with wild type, with breaks observed at the positions for *hns* and *stpA*. These small sections each align to the opposite position, showing the intended reciprocal exchanges were correctly carried out and were detected by sequencing. Vertical red lines indicate the borders of contigs used for genome assembly with both *hns* and *stpA* embedded within large contigs.

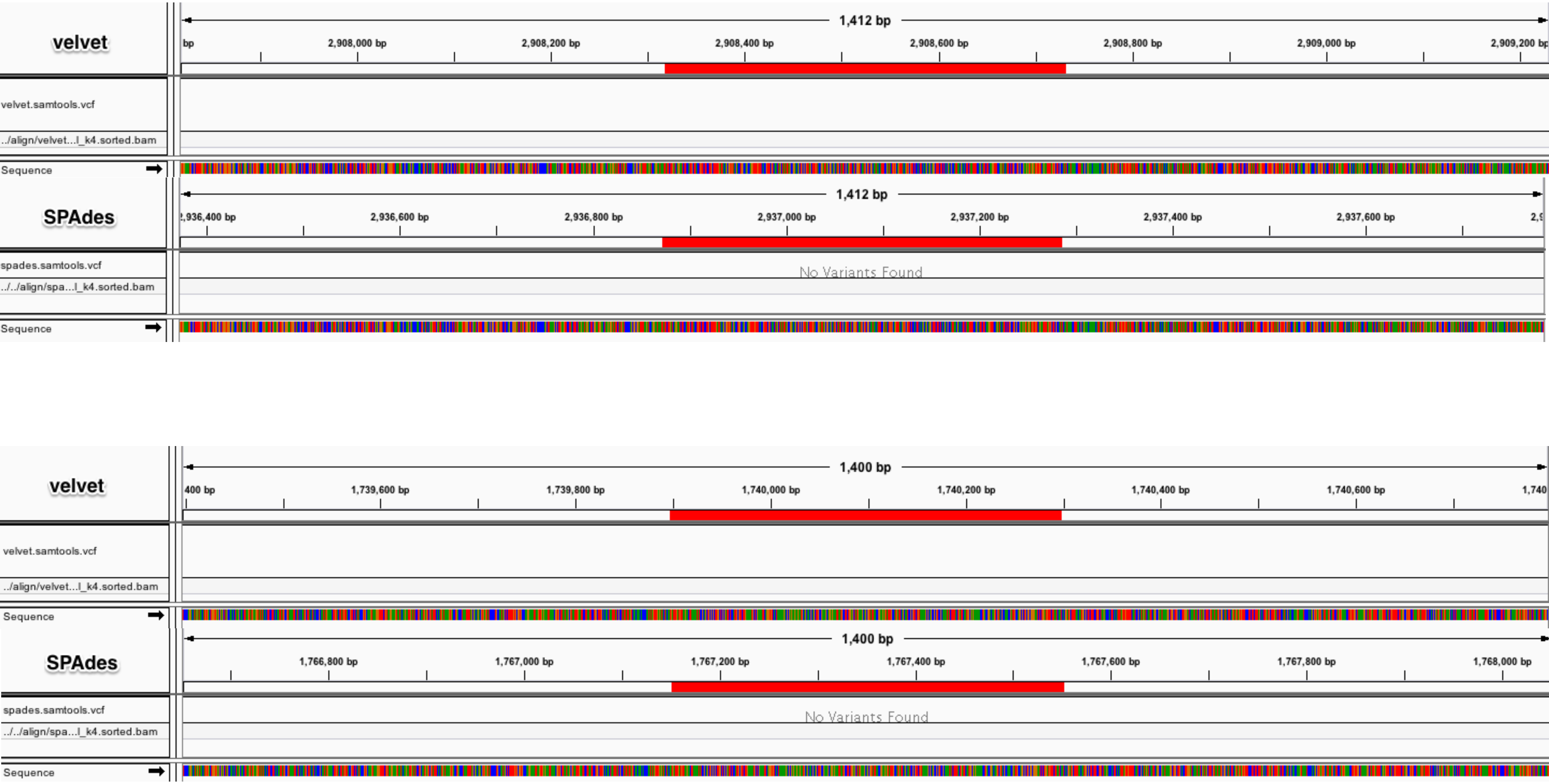


Figure S2| Visualization of the regions adjoining *hns* (top two tracks) and *stpA* (bottom two tracks) in SL1344RX shows no DNA sequence variants were found in either *de novo* assembly.

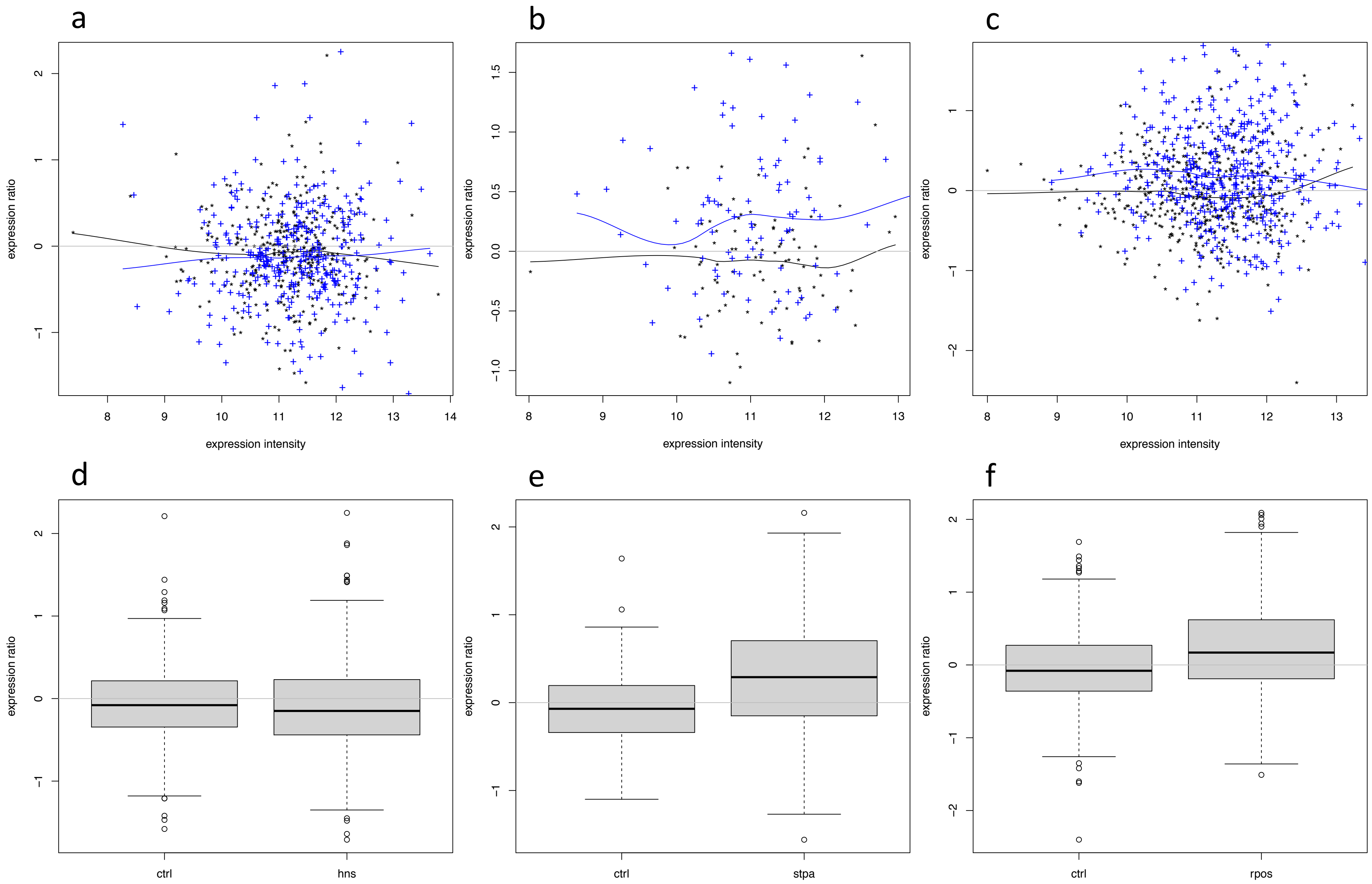


Fig S3| Sample MA-plots (a – c) and corresponding boxplots (d – f) for H-NS (a and d), StpA (b and e) and RpoS (c and f) regulated genes. H-NS, StpA or RpoS regulated genes (blue stars) were plotted together with an equivalent number of control non -H-NS, -StpA or –RpoS regulated genes (black stars). Loess curves displaying the average value across sample (blue line) and control (black line) datasets were fitted. Ten separate control gene sets were used for comparison with each regulon and significant changes in gene expression ($P < 0.01$) identified by t-test. Significant changes in expression of both the StpA and RpoS regulons were observed in each of ten comparisons but no significant changes were observed across the H-NS regulon.

**Mass spectrometric analysis**

Mutant and wild type cells were harvested as for the Western blots and lysed with bead beating. Proteins were extracted using acetone precipitation. After resolubilization in 50 mM ammonium bicarbonate buffer the protein sample was digested with trypsin (Sigma-Aldrich). The resulting peptides were analysed with a Synapt G2 HDMS (Waters) coupled to a Nanoacquity (Waters) nano-LC with an Acquity UPLC T3HSS column (75 mm x 200 mm). Several runs in sensitive as well as resolution mode (scan length 0.7-1.2 s) were performed using data-independent-acquisition (MSE) with leu-enkephaline as lockspray for mass correction. Spectra were subsequently analysed with the ProteinLynx Global Server version 3.02 (Waters). Searches were allowed to reiterate until a false discovery rate of 4% was reached. Identified masses corresponding to StpA were further used to create an inclusion list for subsequent data-dependent analysis.

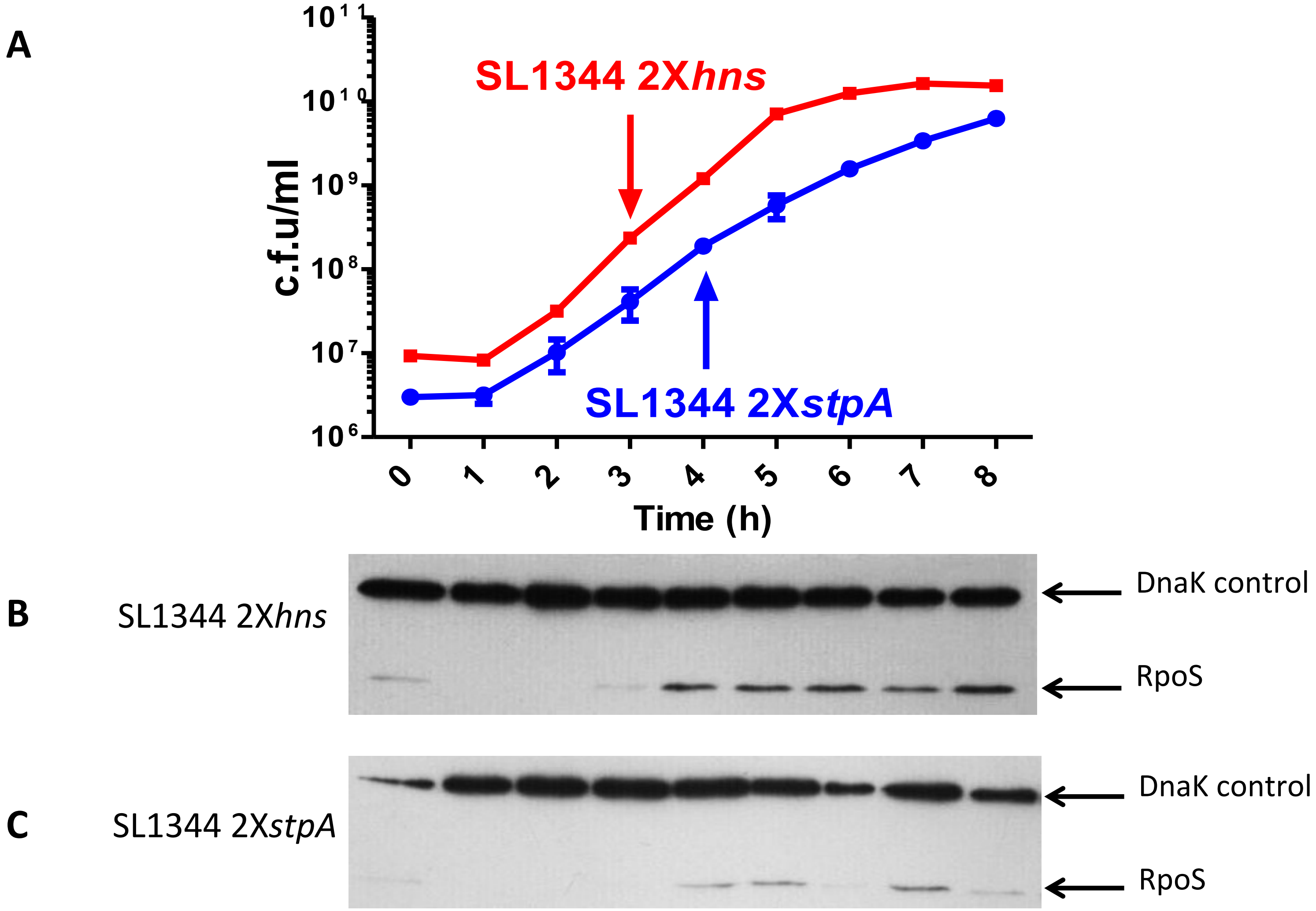


Figure S4| Expression of the RpoS sigma factor in SL1344 2X*stpA* and in SL1344 2X*hns* as a function of growth. The growth cycles of SL1344 2X*stpA* and SL1344 2X*hns* were determined by colony counts (a). RpoS expression was monitored by Western blotting and was first detected after 3 h and 4 h of growth in SL1344 2X*hns* (b) and SL1344 2X*stpA* (c), respectively (n=1).

Table S1 | High stringency (>95% variant read frequency) and low stringency (>45% variant) SNPs identified in SL1344RX.

| Gene | Position | Variant (Ref -> RX) | Variant frequency | Coverage | Effect | Gene product |
|---|---|---|---|---|---|---|
| *manX* | 635,606 | SNP (T -> A) | 100% | 180-fold | Substitution (E->V) | mannose specific permease |
| *menC* | 2,411,272 | SNP (T -> C) | 100% | 213-fold | Synonymous | O-succinylbenzoate synthase |

## Table S2 | Differentially expressed genes in SL1344RX

| STM Identifier | Gene Name | $Log_2$ Ratio | P-value | EMBL assigned function |
|---|---|---|---|---|
| STM1729 | *yciF* | 5.10 | 2.85E-07 | conserved hypothetical protein |
| STM1730 | *yciE* | 4.24 | 1.26E-07 | conserved hypothetical protein |
| STM1267 | | 3.14 | 1.27E-04 | hypothetical protein |
| STM4561 | *osmY* | 3.13 | 2.09E-05 | Putative periplasmic protein |
| STM0465 | *ybaY* | 2.93 | 7.43E-04 | conserved hypothetical lipoprotein |
| STM2795 | *ygaU* | 2.69 | 2.30E-03 | conserved hypothetical protein |
| STM0831 | *dps* | 2.68 | 2.88E-03 | DNA protection during starvation protein |
| STM0562 | *cusA* | 2.66 | 8.14E-02 | cation efflux protein (pseudogene) |
| - | *sopE* | 2.63 | 3.31E-02 | Type III secretion system effector protein |
| STM1119 | *wrbA* | 2.46 | 1.24E-02 | trp repressor binding protein |
| STM1731 | *katN* | 2.44 | 1.59E-03 | catalase |
| STM2164 | *yehY* | 2.28 | 4.29E-03 | hypothetical permease transmembrane component |
| STM1091 | *sopB* | 2.25 | 1.48E-02 | Type III secretion system effector protein. |
| STM2884 | *sipC* | 2.21 | 5.36E-02 | Pathogenicity island 1 Type III secretion system effector protein |
| STM1929 | *otsB* | 2.17 | 8.09E-04 | trehalose phosphatase |
| STM1565 | *rpsV* | 2.16 | 1.74E-03 | 30S ribosomal protein S22 |
| STM1500 | *ynfD* | 2.09 | 1.35E-01 | hypothetical exported protein |
| STM2883 | *sipD* | 2.08 | 5.82E-02 | SPI-1 Type III secretion system translocon protein |
| STM2474 | *tktB* | 2.06 | 2.63E-03 | transketolase 2 |
| STM3571 | *ftsY* | 2.02 | 1.49E-02 | cell division protein |
| STM2577 | *acpS* | 2.01 | 9.21E-03 | Holo-[acyl-carrier-protein] synthase |
| STM1311 | *osmE* | 2.01 | 2.48E-03 | osmotically inducible lipoprotein E precursor |
| STM0759 | *ybgS* | 1.98 | 4.68E-03 | hypothetical exported protein |
| STM0326 | *dhaF* | -2.01 | 1.21E-02 | glycerol dehydratase reactivation factor large subunit (pseudogene) |
| STM4392 | *priB* | -2.38 | 4.49E-02 | primosomal replication protein N |
| STM2434 | | -2.40 | 1.88E-02 | hypothetical protein |

Table S3 | Differentially expressed RpoS regulated genes in SL1344RX

| STM Identifier | Gene Name | Fold Change | P-value | EMBL assigned function |
|---|---|---|---|---|
| STM1729 | *yciF* | 5.10 | 2.85E-07 | conserved hypothetical protein |
| STM1730 | *yciE* | 4.24 | 1.26E-07 | conserved hypothetical protein |
| STM4561 | *osmY* | 3.13 | 2.09E-05 | Putative periplasmic protein |
| STM2795 | *ygaU* | 2.69 | 2.30E-03 | conserved hypothetical protein |
| STM0831 | *dps* | 2.68 | 2.88E-03 | DNA protection during starvation protein |
| STM1119 | *wrbA* | 2.46 | 1.24E-02 | trp repressor binding protein |
| STM1731 | *katN* | 2.44 | 1.59E-03 | catalase |
| STM2164 | *yehY* | 2.28 | 4.29E-03 | hypothetical permease transmembrane component |
| STM1929 | *otsB* | 2.17 | 8.09E-04 | trehalose phosphatase |
| STM1565 | *rpsV* | 2.16 | 1.74E-03 | 30S ribosomal protein S22 |
| STM2474 | *tktB* | 2.06 | 2.63E-03 | transketolase 2 |
| STM1311 | *osmE* | 2.01 | 2.48E-03 | osmotically inducible lipoprotein E precursor |
| STM0465 | *ybaY* | 2.93 | 7.43E-04 | conserved hypothetical lipoprotein |

Table S4 | Bacterial Strains and plasmids

| **Strain or Plasmid** | **Relevant characteristic** | **Resistance** | **Source/reference** |
|---|---|---|---|
| ***S.* Typhimurium** | | | |
| SL1344 | *rpsL hisG46* | | Hoiseth and Stocker(1981) |
| *hns*-FLAG | *hns*::3xFLAG tag | $Km^R$ | This work |
| *stpA*-FLAG | *stpA*::3x FLAG tag | $Km^R$ | This work |
| 2X *hns* | $P_{stpA}$-*hns* | $Km^R$ | This work |
| 2X *stpA* | $P_{hns}$-*stpA* | $Tc^R$ | This work |
| SL1344RX | $P_{stpA}$-*hns* $P_{hns}$-*stpA* | $Km^R$ $Tc^R$ | This work |
| SL1344RX *hns*-FLAG | $P_{stpA}$-*hns*-3xFLAG $P_{hns}$-*stpA* | $Km^R$ $Tc^R$ | This work |
| SL1344RX *stpA*-FLAG | $P_{stpA}$-*hns* $P_{hns}$-*stpA*-3xFLAG | $Km^R$ $Tc^R$ | This work |
| pKD46 | λ genes *gam*, *bet* and *exo* under the control of the arabinose inducible pBAD promoter | $Amp^R$ | Datsenko and Wanner (2000) |
| pSUB11 | pGP704 derivative containing the $Km^R$ cassette with flanking FRT sites from pKD4 and upstream 3xFLAG-tag sequence | $Km^R$ | Uzzau *et al* (2001) |

$Km^R$ = Kanamycin resistant, $Tet^R$ = Tetracycline resistant, $Amp^R$ = Ampicillin resistant.

Table S5 | Oligonucleotide primer sequences

| | |
|---|---|
| **hns.RT.fw** | 5'-gca caa ctg aat tta tcg ct-3' |
| **hns.RT.rv** | 5'-cat tct ctt gcc tgc gca-3' |
| **stpA.RT.fw** | 5'-aat cgc ata caa tac cgc-3' |
| **stpA.RT.rv** | 5'-ggc tcg cga att ctc cat tg-3' |
| **hns.flag.fw** | 5'-aga aca agg taa gca act gga aga ttt cct gat caa gga aga cta caa aga cca tga cgg-3' |
| **hns.flag.rv** | 5'-aaa aat ccc gcc agc ggc ggg att tta agc atc cag gaa gca tat gaa tat cct cct tag-3' |
| **stpA.flag.fw** | 5'-ggc gct ggc ggc ggg gaa atc tct gga tga ttt ctt aat cga cta caa aga cca tga cgg-3' |
| **stpA.flag.rv** | 5'-cgg att aga aaa aca act taa atg tga aag tgg gtc tta aca tat gaa tat cct cct tag-3' |
| **stpA.tet.fw** | 5'-gga tag ctt tta aca gat ggc gct tcg ttt cct gtc tta aga ccc act ttc aca tt-3' |
| **stpA.tet.rv** | 5'-atg agc atg gcg caa cgt tcc ctg agt ccc ggt taa cta agc act tgt ctc ctg-3' |
| **hns.kan.fw** | 5'-ctt cct gga tgc tta aaa tcc cgc cgc tgg cgg gat cat atg aat atc ctc ctt ag-3' |
| **hns.kan.rv** | 5'-gcc tgg ggt cgt cag cgg aga act cag gca aaa aaa gac tac aaa gac cat gac gg-3' |
| **stpA.int.fw** | 5'- aat agt ttt ttg ttt tct gcg tta aaa ggt ttt tat tga tat gag cga agc act taa aat-3' |
| **stpA.int.rv** | 5'-gac agg aaa cga agc gcc atc tgt taa aag cta tcc gtg att acg ccc cgc cct gcc act-3' |
| **hns.int.fw** | 5'-gct caa caa acc acc cca ata taa gtt tga gat tac tac aat gaa ttt gat gtt aca gaa-3' |
| **hns.int.rv** | 5'-atc ccg cca gcg gcg gga ttt taa gca tcc agg aag taa att acg ccc cgc cct gcc act-3' |

RT = RT-qPCR, flag = 3X FLAG-tag epitope tagging, tet = integration of *tetRA*, kan = integration of *neoR*, int = integration at *stpA* or *hns* loci
